# From a Point Cloud to a Simulation Model—Bayesian Segmentation and Entropy Based Uncertainty Estimation for 3D Modelling

**DOI:** 10.3390/e23030301

**Published:** 2021-03-03

**Authors:** Christina Petschnigg, Markus Spitzner, Lucas Weitzendorf, Jürgen Pilz

**Affiliations:** 1 BMW Group, Department of Factory Planning, Knorrstraße 147, 80788 Munich, Germany; Markus.Spitzner@bmw.de (M.S.); Lucas.Weitzendorf@bmw.de (L.W.); 2 Department of Statistics, Alpen-Adria-University Klagenfurt, Universitätsstraße 65-67, 9020 Klagenfurt, Austria; Juergen.Pilz@aau.at

**Keywords:** factory planning, factory simulation, digital factory, Bayesian deep learning, uncertainty estimation, point clouds, photogrammetry

## Abstract

The 3D modelling of indoor environments and the generation of process simulations play an important role in factory and assembly planning. In brownfield planning cases, existing data are often outdated and incomplete especially for older plants, which were mostly planned in 2D. Thus, current environment models cannot be generated directly on the basis of existing data and a holistic approach on how to build such a factory model in a highly automated fashion is mostly non-existent. Major steps in generating an environment model of a production plant include data collection, data pre-processing and object identification as well as pose estimation. In this work, we elaborate on a methodical modelling approach, which starts with the digitalization of large-scale indoor environments and ends with the generation of a static environment or simulation model. The object identification step is realized using a Bayesian neural network capable of point cloud segmentation. We elaborate on the impact of the uncertainty information estimated by a Bayesian segmentation framework on the accuracy of the generated environment model. The steps of data collection and point cloud segmentation as well as the resulting model accuracy are evaluated on a real-world data set collected at the assembly line of a large-scale automotive production plant. The Bayesian segmentation network clearly surpasses the performance of the frequentist baseline and allows us to considerably increase the accuracy of the model placement in a simulation scene.

## 1. Introduction

There is no common definition of factory planning. However, a widely used description comes from the Association of German Engineers (VDI), which defines factory planning as “systematic, objective-oriented process for planning a factory, structured into a sequence of phases, each of which is dependent on the preceding phase, and makes use of particular methods and tools, and extending from the setting of objectives to the start of production” [1]. Four major types of factory planning scenarios are distinguished, namely development or greenfield planning, replanning or brownfield planning, dismantling and revitalization.

### 1.1. Greenfield and Brownfield Planning

The most common planning scenario is brownfield planning followed by greenfield planning. Greenfield and brownfield planning are distinct processes that have different aims and requirements. Thus, different considerations are needed to achieve the respective planning goals. Greenfield planning refers to planning a completely new plant with a new building, production line and processes. The generation of three-dimensional (3D) simulations and the introduction of changes to the virtual production system are relatively easy as new plans fulfil current requirements of factory planning including 3D building plans, 3D layouts and computer aided design (CAD) models of the facility, machinery and products. Further, new technologies and the need for digitalization are taken into account during planning. For instance, cameras and targets are added to the plants for later digitalization. Due to the availability of a digital model of the production system, planners are able to detect planning mistakes mostly in the virtual world before the actual implementation. Brownfield planning in contrast refers to the renovation or reorganization of an existing plant. This task is much more difficult compared to greenfield planning as existing layouts are often outdated and only available in two-dimensional (2D) form. These plans hide substantial depth information, which is especially important when tasks are related to the introduction of new machinery or process steps. Due to the enormous area of assembly plants, which easily reaches up to several thousands of square metres, various problems arise. The determination of the as-is state of these plants is difficult, as layouts are often outdated and do not reflect all the renovations of the facility and the production line. The spatial requirements for a new planning case can hardly be determined by manual measurements due to the vast area of the plants. Further, certain technological constraints have to be accepted or modified with great effort. For instance, in older plants cabling, out-lets and interfaces for newer digitalization technologies are limited or even non-existent, which makes them difficult to digitalize. As the share of virtual planning is small compared to analogue planning many planning mistakes can only be detected during implementation, which is costly. The major differences between greenfield and brownfield planning are summarized in Table 1.

In order to cope with the challenges arising in brownfield planning scenarios, virtual planning undoubtedly provides great potential in terms of planning efficiency and accuracy. It has many advantages over analogue planning, for example, virtual commissioning and virtual correction of mistakes. The highest economic benefit is the detection and correction of planning mistakes in early project phases—ideally before their actual implementation [2]. Further, factory planners may inspect overseas sites in a purely virtual environment to discuss modifications or other planning related tasks. A virtual 3D copy of the real plant can be transferred to virtual reality supported environments or simpler simulation engines, which usually provide the possibility of multi-user meetings. In the long run this saves a considerable amount of travel time and cost. Ideally, solely 3D models and corresponding simulations of factory buildings, inventory assets, product information and process steps are the basis for implementing assembly reorganizations including the introduction of new or the modification of existing processes.

### 1.2. Factory Planning and Digitalization

Until the early 20th century, the manufacturing industry was apt to mass production mainly requiring robustness to interruptions. Since that time, production systems had to change fundamentally due to shorter product lifecycles, decreasing lot sizes and progressive product customization [3]. The factory lifecycle accompanies the factory from its beginnings, that is, the initial planning, to its end of life. It is mainly determined by the shortening product and process lifecycles. The factory lifecycle can be split into five distinct phases, namely, development, construction, start-up, operation and dismantling [4].

According to the factory lifecycle, the factory planning process is part of the development, as well as the operation phase, of greenfield and brownfield planning, respectively. The factory planning process itself can be divided into another seven distinct phases, which are described in more detail in the VDI guideline 5200 [1]. The first two phases aim to describe the objectives and the collection of preliminary planning data. During the third and fourth phase the planning concepts for structural elements are specified until a realizable status is reached. The final phases prepare for the realization. They supervise and support the factory start-up. All of these phases are accompanied by project management. The seven phases of the factory planning process are part of any greenfield or brownfield planning project. With this phase model the VDI established a quasi-standard for factory planning, which is adopted by many manufacturing companies. Thus, the proposed techniques prove to be well elaborate and reliable. Undoubtedly, the availability of current 3D factory data and simulation models facilitates the realization of any of these phases in greenfield as well as brownfield planning. However, in this work we focus on brownfield planning and the acquisition of current models mainly for the structural part, that is, the third and fourth factory planning phase.

In order to arrive at a current digital model of a production site, there are several hurdles to overcome. The first and at the same time one of the most challenging problems is the collection of current data in the plants, each of which extends over a huge area. In recent years laser scanning [5] and photogrammetry [6] have become popular measures for indoor digitalization, generating 3D point clouds of the environment. As soon as the necessary 3D data is available, it has to be pre-processed in order to be useful for factory planning. The pre-processing includes the fusion of data collected by different sensors and data cleaning. For the mere aim of a rough visualization of the environment, the availability of 3D point clouds is sufficient. Promising results for surface reconstruction on the basis of point clouds are presented in [7,8]. However, these approaches do not aim at reconstructing an industrial environment but focus on the meshing process. In order to achieve photorealistic reconstruction results, further manual efforts are needed. The generation of an environment or simulation model consisting of individual objects requires the identification and the localization of these objects within the point cloud, which can be tackled by object detection networks that directly perform pose estimation or a segmentation framework, where object poses are estimated on the basis of the segmented point cloud. Both of these approaches are discussed and contrasted with respect to their advantages and disadvantages in Section 2.1. The availability of CAD models of the objects of interest is an advantage for model generation, as many simulation tasks cannot be carried out solely on the basis of point clouds. Raw point clouds are subject to occlusions, which cause holes within the point cloud. Therefore, tasks like collision checking are potentially not reliable. Further, working with an unsegmented point cloud has the disadvantage of not being able to distinguish between different objects automatically. They have to be selected manually, which makes their displacement or removal in a scene inconvenient. Yet, replacing CAD models by reference point clouds is possible although some of the informative value of the resulting simulation can get lost.

### 1.3. Contributions of the Paper

**Figure 1:**
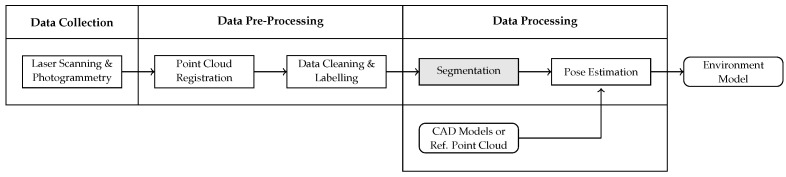
Process diagram of generating an environment model on the basis of a point cloud. The process steps are displayed in rectangle shaped boxes. Input and output data are represented by boxes with rounded corners. The segmentation step is highlighted as this step is explained in more detail in [10].

In this work, we propose an approach to reconstruct an existing industrial production plant including building, product and process related structures based on a 3D point cloud. We determine the impact of the uncertainty information gained by a Bayesian neural network on the reconstruction results. Currently, this is still an open research question with hardly any literature focusing on the requirements of industrial applications. We focus on the brownfield planning case and propose a holistic and methodical modelling approach based on [9] for the automated generation of an environment model in a simulation engine starting from the collection of a raw point cloud of a manufacturing plant. Initially, a data collection methodology based on 3D laser scanning and photogrammetry is proposed, which enables us to collect a data set at a German automotive OEM in order to thoroughly test our remaining workflow steps. The digitalization process yields a 3D point cloud of the factory environment. Objects therein are identified using a segmentation approach. We incorporate the information on the segmentation uncertainty and investigate its impact on the resulting model. As Bayesian methods provide a mathematically sound framework for uncertainty quantification, we apply the Bayesian neural network (BNN) proposed in our previous work [10]. The BNN is capable of 3D semantic segmentation and thus allows us to partition the point set into meaningful subsets that can be provided to the respective departments for further processing. Aside from neural networks there are other methods for point cloud segmentation, for example, octree [11], superpoint graphs [12] or hierarchical clustering [13] based techniques. However, these methods are not capable of expressing uncertainty in the predictions. Therefore, we decide to apply a deep neural network trained in a Bayesian way. The segmented output point cloud is used to determine the number of objects per scene as well as their pose relative to a predefined zero point. These steps are carried out by the application of clustering and point cloud registration algorithms. To this end, either CAD models or reference point clouds of the objects of interest are required. The proposed modelling approach for the generation of a static simulation model is depicted in Figure 1.

The proposed framework is part of an industrial prototype that aims at the reconstruction of automotive assembly plants on the basis of point cloud data. However, this approach can well be applied to any indoor industrial or office areas. In summary, the contributions of this paper are:

- Framework: We describe a comprehensive and methodical modelling approach starting with the digitalization of large-scale industrial environments using laser scans and photogrammetry and ending with the generation of a static environment model.
- Experiment: We evaluate the quality of factory digitalization in terms of the accuracy, completeness and point density of the resulting point cloud. Further, the accuracy of the final environmental model is evaluated. The segmentation model used in this work is presented and evaluated in [10].
- Potential: We provide an estimation of the economic potential of automated factory digitalization as well as simulation model generation for a number of exemplary production plants.

The remainder of this paper is structured as follows—in Section 2, environment modelling approaches based on point clouds are discussed as well as the notion of uncertainty in predictive models. Further, the collection and pre-processing of our automotive factory data set is described. Section 3 focuses on the applied Bayesian segmentation network and describes the methodologies for pose estimation and simulation model generation. The evaluation of the proposed steps is presented in Section 4 including the description of the data set used. The complete simulation model generation approach is evaluated by processing our own automotive factory data set. Finally, Section 5 provides a discussion of the proposed approach as well as its economic potential before concluding the paper.

## 2. Data Modelling

The proposed approach relies on the availability of point cloud data for 3D modelling. In the sequel point cloud processing frameworks as well as solutions to environment modelling based on point clouds are discussed. Further, Bayesian methods and the uncertainty definitions applied within this paper are discussed as well as the collection and pre-processing of our automotive factory data set.

### 2.1. Point Clouds and Environment Modelling

Learning frameworks on 3D point clouds have to cope with the unordered and irregular structure of the inputs. Some of these frameworks include [14,15,16]. Several strategies exist to transform point clouds to a more regular representation, namely voxelization or the projection to the 2D space. In the former case a volumetric grid is created that represents the point cloud in a regular format. Different deep learning approaches, which use voxelized point clouds as an input, are discussed in [17,18,19]. In the latter case, the input point cloud is projected to the 2D space under varying viewports [20,21,22]. The transformation to a regular data structure has many advantages like the possibility to apply either 2D or 3D convolutions with their elaborate kernel optimizations. However, voxelization unnecessarily expands the data volume as empty areas in the point cloud are still represented by voxels. Further, dense areas of the point cloud are displayed in a lower resolution by the voxel grid, which entails an information loss [23] and truncation errors [15]. The transformation of 3D point clouds to 2D images can cause the loss of structural information embedded in the higher dimensional space. Additionally, a high number of viewports need to be considered in order to represent the information encoded within a point cloud in a number of images [23]. Thus, we decide to work directly with the raw and unordered point cloud as an input for the segmentation network.

Generally, two major methodologies on environment reconstruction exist, namely data- and model-driven approaches [24]. The former directly estimate features from the data, while the latter take into account prior knowledge to choose a suitable model from a library and fit it to the data. As we do not have a complete catalogue or library of existing models and only limited prior knowledge on the interior of far-away plants is available, data-driven approaches are reviewed in more detail in the sequel. Point clouds are often used as data source for environment modelling. Different objects in the point cloud need to be identified, which can be solved using various statistical or machine learning techniques. Data-driven techniques are heavily used for modelling archaeological sites [25]. Further, they are used in architecture for building modelling, however, they have rather simple applications like the extraction of windows and doors in façades [26,27] or the creation of floor plans [28]. In [29] an approach that reconstructs and interprets indoor areas is presented. Similarly to the previous methods, the reconstructed objects are limited to the floor, the ceiling, walls and doors. Aside from indoor applications, in [30] an outdoor point cloud is segmented by clustering the point cloud of an urban area into a number of plane shaped groups. Based on these surfaces, building models are reconstructed. Another outdoor building model is generated using oblique areal images as well as edge and height information of façades [31]. However, none of these frameworks has an industrial application requiring a detailed environment model including product, process and building information.

Deep semantic segmentation is a way of assigning a distinct class label to each point in the point cloud, thus, allowing the differentiation of objects in the point set. In our modelling approach, we identify product, process and building related objects and estimate their 9DoF pose, that is, position, rotation and scale with respect to some predefined coordinate origin, based on a segmented point cloud.

Aside from segmentation, object detection and pose estimation can be performed directly on the point cloud. The Scan2CAD framework directly estimates the 6DoF CAD model alignment, that is, position and rotation, of eight household objects on the basis of a voxelized point cloud and the respective CAD models [32]. An extension of this framework, which is capable of 9DoF pose estimation on the same data set, is discussed in [33]. Apart from deep neural networks a CAD model alignment framework based on global descriptors extracted by the Viewpoint Feature Histogram approach [34] is described in [35]. Direct object detection and estimation of the CAD model alignment on the basis of point clouds can of course be used for environment modelling and simulation model generation. It has the advantage of only one classifier to be trained for generating an environment model. However, it requires the availability of the CAD models of all objects of interest. The prior segmentation of a point cloud with a subsequent pose estimation step allows us to model objects where CAD models are not available on the fly. Further, meaningful partitions of the point cloud can be generated, that is, building, assembly, logistics and product related point sets. These subsets can be provided to the respective departments that only process the necessary parts of the point cloud, thus, reducing the storage and computational efforts considerably. Therefore, our approach of point cloud segmentation followed by a separate pose estimation step addresses the factory planning use case in a holistic way, which is not covered by previous works.

### 2.2. Bayesian Neural Networks and Uncertainty Definition

Bayesian neural networks in contrast to frequentist or classic neural networks take on the Bayesian interpretation of probability. They place a prior distribution over all the network parameters including weights and biases, instead of considering them as point estimates. Thus, a prior distribution needs to be defined for all the network parameters. The posterior distribution can be calculated using Bayes’ theorem after each batch of training data. However, the direct application of Bayes’ theorem is difficult as a generally intractable integral needs to be solved. Several techniques to approximate the posterior exist, for example, variational inference [36], Markov Chain Monte Carlo approaches [37,38,39], Hamiltonian Monte Carlo algorithms [40] and Integrated Nested Laplace Approximations [41]. For training efficiency the generally fastest method of variational inference is used in the following.

As already mentioned, BNNs allow the estimation of uncertainty in the network outputs, which can be realized using a number of different approaches. Many works distinguish between uncertainty inherent to the data and model related uncertainty. These are called aleatoric and epistemic uncertainty, respectively [42]. The total uncertainty in a network prediction, which is referred to as predictive uncertainty, can be interpreted as the sum of aleatoric and epistemic uncertainty. Such a split of uncertainty into data and model related uncertainty is useful as it allows us to grasp the amount of uncertainty that can be reduced by further model optimization and the amount of uncertainty, which is inherent to the data. One way of estimating uncertainty in a BNN is based on entropy [43]. Precisely, predictive uncertainty $U_{pred}$ can be modelled as the entropy H inherent to the predictive network outputs, that is, $U_{pred}\;=\;H\left[y^{🟉}|{\underline{x}}^{🟉},\underline{w}\right]$, where $\left({\underline{x}}^{🟉},y^{🟉}\right)$ is an unseen input with its corresponding label and $\underline{w}$ corresponds to the network parameters. In practical applications the marginalization over the weight samples drawn from an approximate variational distribution yields $U_{pred}$,

(1) $$U_{pred}\;\approx \;-\sum_{y^{🟉}\in \left\{1,\cdots ,m\right\}}\left(\frac{1}{K}\sum_{k=1}^{K}p\left(y^{🟉}|{\underline{x}}^{🟉},\;{\underline{w}}^{k}\right)\right)\cdot log\left(\frac{1}{K}\sum_{k=1}^{K}p\left(y^{🟉}|{\underline{x}}^{🟉},\;{\underline{w}}^{k}\right)\right).$$

In Equation (1), $p(y^{🟉}|{\underline{x}}^{🟉},\;{\underline{w}}^{k})$ represents the predictive network output and ${\underline{w}}^{k}$ is the *k*-th weight sample of the variational distribution $q_{\underline{\varphi }}$, which is described in more detail in Section 3.1.2. In total, K∈N weight samples are drawn from the variational distribution. The average entropy over all weight samples ${\underline{w}}^{k}$ corresponds to the aleatoric uncertainty,

(2) $$U_{alea}\;=\;E\left[H\left[y^{🟉}|{\underline{x}}^{🟉},{\underline{w}}^{k}\right]\right]\;\approx \;-\frac{1}{K}\sum_{k=1}^{K}\sum_{y^{🟉}\in \left\{1,\cdots ,m\right\}}p\left(y^{🟉}|{\underline{x}}^{🟉},{\underline{w}}^{k}\right)\cdot log\left(p\left(y^{🟉}|{\underline{x}}^{🟉},{\underline{w}}^{k}\right)\right).$$

Epistemic uncertainty $U_{ep}$ is calculated as the difference of predictive and aleatoric uncertainty, that is, $U_{ep}=U_{pred}-U_{alea}$.

Further ways of uncertainty quantification include the variance of the predictive network outputs and the estimation of (95 %-)credible intervals [44]. In the former case the predictive variance is estimated empirically using the unbiased estimator for the variance. Any prediction for which the predictive variance exceeds a certain threshold, for example, the mean variance plus two sigma, is considered uncertain. In the latter case, credible intervals are calculated for each class. If the credible interval of the predicted class overlaps with any other class’ credible interval the prediction is considered uncertain. In [10] all of these methods for uncertainty quantification are contrasted.

### 2.3. Data Collection

In recent years, laser scanning became increasingly popular for the purpose of indoor and outdoor digitalization [45]. The trend was fostered mostly by the recent advances in the scanning technology, increasing storage capacity and decreasing equipment cost. As a rule of thumb, stationary laser scanners produce a more accurate point cloud than mobile ones as they stand still during digitalization. Further, stationary laser scanners usually have a longer range than mobile ones, which is insignificant in the plane but becomes important when measuring heights. However, stationary laser scanners produce point clouds with a high point density close to the device, which is decreasing over distance, while mobile laser scanners capture the environment with a lower but more homogeneous point density. Further, stationary scanners take a longer time to be set up and capture their surroundings with as few occlusions as possible [46]. A comparison of stationary and mobile laser scanning is depicted in Table 2.

In industrial manufacturing plants, where high-quality data of the as-is state of production plants are often missing, regular digitalization activities are needed. As already mentioned, brownfield planning scenarios lack current factory data, sometimes not even 3D information is available. Further, not all renovation works are properly reflected in the layouts. Thus, over the years a plant layout can be up-to-date in some places and outdated in others, with 3D information being available only for some parts of the plant. Therefore, it is very important to find a way of methodical and periodically recurrent data collection to ensure the quality and timeliness of the plant layouts, CAD models and construction drawings of production and logistics equipment.

**Figure 2:**
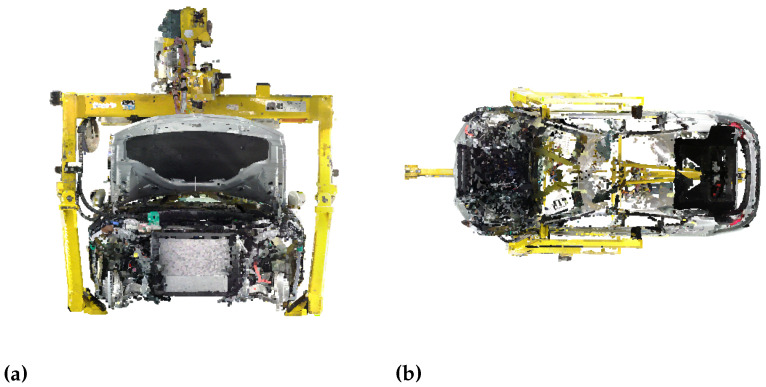
Impact of occlusions on a point cloud. (**a**) The point cloud of the car looks complete at the surface facing the laser scanner. (**b**) The bottom view of the car reveals the empty interior due to occlusions. The roof of the car is incomplete due to the reflection of the impinging laser beams.

Stationary laser scanning is a popular method of digitalizing production plants. In order to reduce the number of stationary scans needed to generate a high-quality point cloud of the factory environment, additional sources of data can be added. For instance, mobile laser scanners or photogrammetry techniques or even both can be used to complement stationary laser scan point clouds. The application of several digitalization measures reduces the number of holes in the resulting point cloud and the point density is increased. Figure 2 displays the impact of occlusions on a point cloud. Figure 2a illustrates a complete point cloud of the front of a car, which is facing the laser scanner. Figure 2b represents the bottom view of the same car. Due to occlusions during the digitalization process, the interior of the car is not captured and thus represented by empty space. The holes in the roof of the car are caused by reflections of the laser beams. In order to generate a complete point cloud, it is therefore important to choose the scan positions carefully. As not all use cases of point clouds require the same resolution and accuracy, it is efficient to carry out base digitalization using mobile scanners while conducting stationary scans only where higher accuracy is really needed.

**Figure 3:**
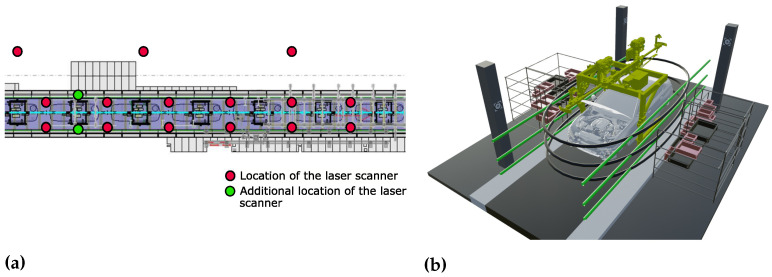
Locations of data collection. (**a**) Laser scanner positions for collecting four (red) and six (green) laser scans per tact. We collect laser scans of the assembly line and the surrounding building to generate our data set. The six laser scan set-up is used for evaluation purposes. (**b**) Concept of taking camera images.

Before starting to digitalize any large-scale environment, it is advisable to place so-called registration targets in the facility in order to facilitate the fusion of different point clouds into one common coordinate system. After placing the targets, the actual digitalization process starts. For the following experiments, we digitalize several tacts of an automotive assembly plant by placing a stationary scanner in different positions on the assembly line and next to it during the production free time. As discussed before, the choice of the scanner positions is important to reduce object occlusions to a minimum, which results in a point cloud with a minimum number of holes. Figure 3a illustrates the scanner positions on a rough sketch of a factory layout for several tacts. In this layout four and six laser scans are marked per assembly tact. The four laser scans, which we use to digitalize the assembly area, are displayed in red and the additional two laser scans are marked in green, which we use for the evaluation of data collection. Further scans are generated outside of the assembly line in order to capture additional facility structures as well as production equipment.

In addition to the laser scans, we roughly take 500 camera images of each tact as well as several hundreds of the surrounding factory area. Prior to taking the images to generate the photogrammetric point cloud, the camera with the respective lens needs to be calibrated in order to correct distortions in the images taken. We use a chequerboard calibration pattern and collect images from different views in order to determine the camera parameters and calibrate the camera with the respective lens. Figure 3b shows the height at which the images are taken. The grey lines represent the height at which inward facing images are taken, that is, images of the assembled vehicle. The green lines indicate the height at which outward facing images are taken, that is, images of the lineside layout.

### 2.4. Data Pre-Processing

The stationary laser scans produce point clouds that have a local coordinate system. The process of generating a single global point cloud out of the local point clouds is called registration. The registration is carried out using so-called registration targets, which are 2D patterns of highly contrasting colors, whose shape is easily located from varying viewpoints. Further distinctive points that are visible in several point clouds can be added manually in the case that registration cannot be completed using the targets alone. This provides a rough registration, which can be improved by the iterative closest point (ICP) algorithm [47].

After the registration of the laser scan point clouds, the information contained in the image data has to be added. In our approach the image data is sorted according to the assembly tact and blurred images are removed, which is automated using a technique based on convolutions [48]. However, the removal of some of the images leaves us with a different number of images per tact, which can influence the resulting point cloud density, see Section 4.2.1. The information included in the remaining images has to be combined with the laser scan data. Due to the high number of reflecting objects within the environment like painted car bodies or metallic machines, the laser scan point cloud and the camera images are fused on image level. Synthetic images are created on the basis of the laser scan point cloud using the approach in [49]. These synthetic images are fused with the camera images using a structure from motion algorithm [50].

After the fused point cloud, which is made up of laser scans and camera images, is generated, it can still be very noisy. A high degree of noise is neither favorable for visualization nor for segmentation and object pose estimation. Thus, noise suppression and outlier detection algorithms are applied, which remove a number of noise points. In order to achieve a suitable degree of noise removal, the point cloud is further cleaned manually by the authors. In the course of this manual cleaning step the data set is labelled as well. For simulation model generation the point cloud needs to be segmented, which requires supervised training with labelled training data. The labelling step is only needed for the initial model training. It is not explicitly part of the workflow once the model is trained, as the model is only evaluated in the subsequent factory planning projects. In this case, the labelling step is not necessarily needed except for a random check of the accuracy. It is important to identify objects that are relevant for factory planning before labelling the data. Relevant classes are mainly those objects that are immovable, fixed to the ground or part of the building as they need to be taken into account during the next renovation project.

## 3. Methods

Our proposed modelling approach relies on various methods including point cloud segmentation to identify the different objects in the scene. Further, object poses are estimated using a clustering and point cloud registration based scheme. Finally, the simulation model can be created in an arbitrary simulation tool. All of these steps are discussed in more detail in the following.

### 3.1. Point Cloud Segmentation

As already discussed, the generated point cloud is segmented in order to identify the different objects in the scene. Therefore, we use the Bayesian segmentation framework, which we proposed in our previous work [10]. In the sequel the mathematical notations and preliminary information on the segmentation step are detailed, followed by a brief description of the applied network.

#### 3.1.1. Notation and Preliminaries

In Section 2.2, we already mentioned that BNNs place a probability distribution over all network parameters that is optimized during network training. In order to describe such a BNN model, the following notations are used throughout the remaining work. The network parameters include the weights and biases of all d∈N network layers, which are denoted by W and B, respectively. The parameters of network layer *i* are represented by $W_{i}$ and $B_{i},\;i\in \left\{1,\cdots ,d\right\}$. The network inputs are given by $X=\{{\underline{x}}_{1},\cdots ,{\underline{x}}_{n}\}$ and the corresponding labels are depicted by $Y=\{y_{1},\cdots ,y_{n}\}$. The variables n∈N and m∈N represent the number of network inputs and the number of classes, respectively. Let further $p(\underline{w})$ denote the prior distribution of the network parameters. The a posteriori distribution of the network parameters after the evaluation of the training examples is given by $p(\underline{w}|Y,X)$ and can be calculated using the Bayes’ theorem. The calculation of the posterior distribution is usually intractable as a result of the high dimensional sample space. Thus, in many cases it is not possible to find an analytical solution for the posterior distribution, however, several approximation techniques exist. The different posterior approximation methods mentioned in Section 2.2 are reviewed in our previous work [10]. Due to efficiency reasons, we decide to use variational inference for posterior approximation. The main idea of variational inference is to approximate the intractable posterior distribution $p(\underline{w}|Y,X)$ with the so-called variational distribution $q_{\underline{\varphi }}\left(\underline{w}\right)$, which is tractable. The variational distribution is parametric and the components of $\underline{\varphi }$ are referred to as the variational parameters. In order to approximate the posterior distribution, the distance between the posterior and the variational distribution has to be minimized. As an evaluation metric, we used the Kullback-Leibler (KL) divergence, which is a popular measure to determine the similarity of two distributions even though it is not a distance metric in the common sense. It neither satisfies the triangle inequality nor is it symmetric. As the KL-divergence cannot be minimized directly, the equivalent optimization problem of minimizing the negative log evidence lower bound is solved [51].

After the variational distribution $q_{\underline{\varphi }}\left(\underline{w}\right)$ is optimized by processing the training samples (X,Y), the class label $y^{🟉}$ of an unseen data point ${\underline{x}}^{🟉}$ can be predicted. The posterior predictive distribution $p(y^{🟉}|{\underline{x}}^{🟉},Y,X)$ expresses the confidence in a label $y^{🟉}$ after observing the input ${\underline{x}}^{🟉}$. It is calculated using

(3) $$p\left(y^{🟉}|{\underline{x}}^{🟉},Y,X\right)\;=\;\int p\left(y^{🟉}|\underline{w},{\underline{x}}^{🟉}\right)p\left(\underline{w}|Y,X\right)\;d\underline{w}.$$

In practice, the unknown posterior in Equation (3) is replaced by the variational distribution and the integral is approximated by Monte Carlo integration, that is,

(4) $$p\left(y^{🟉}|{\underline{x}}^{🟉},Y,X\right)\;\approx \;\frac{1}{K}\sum_{k=1}^{K}f\left({\underline{x}}^{🟉},{\underline{w}}^{k}\right),\;\mathrm{with}\;{\underline{w}}^{k}\underset{i.i.d.}{∼}q_{\underline{\varphi }}\left(\underline{w}\right),$$

where *f* denotes a forward pass through the network and K∈N corresponds to the number of Monte Carlo weight samples ${\underline{w}}^{k}$ drawn from the variational distribution. The predicted class ${\hat{y}}^{🟉}$ is calculated using

(5) $${\hat{y}}^{🟉}\;=\;\underset{y^{🟉}\in \left\{1,\cdots ,m\right\}}{\;\arg\;\max\;\;}p\left(y^{🟉}|{\underline{x}}^{🟉},Y,X\right).$$

#### 3.1.2. Bayesian Segmentation Network

The network we use for the subsequent evaluations is described in more detail in our previous work [10]. The variational distribution is based on the one proposed in [44]. We define the random weights $W_{i}:=\left(W_{i1},\cdots ,W_{ik_{i}}\right)$ and biases $B_{i}:=\left(B_{i1},\cdots ,B_{ik_{i}^{\prime }}\right)$ of the *i*-th network layer, where $k_{i}\in N$ denotes the number of weights and $k_{i}^{\prime }\in N$ the number of biases of layer i,i∈{1,⋯,d}. The network parameters are defined according to

$$\begin{array}{ccc} \tau_{wi} & := & log(1+exp\left(\delta_{wi}\right))\\ W_{i} & := & {\underline{\mu }}_{wi}⊙\left({\underline{1}}_{k_{i}}+\tau_{wi}{\underline{\varepsilon }}_{wi}\right)\\ \tau_{bi} & := & log(1+exp\left(\delta_{bi}\right))\\ B_{i} & := & {\underline{\mu }}_{bi}⊙\left({\underline{1}}_{k_{i}^{\prime }}+\tau_{bi}{\underline{\varepsilon }}_{bi}\right), \end{array}$$

where ${\underline{1}}_{k}$ is the *k*-dimensional vector consisting of all ones and ⊙ is the Hadamard or elementwise product. The variational parameters are represented by ${\underline{\mu }}_{wi}\in R^{k_{i}}$, ${\underline{\mu }}_{bi}\in R^{k_{i}^{\prime }}$, i∈{1,⋯,d} and $\delta_{wi},\;\delta_{bi}\in R$. We choose $\varepsilon_{wi}\in R^{k_{i}}$ and $\varepsilon_{bi}\in R^{k_{i}^{\prime }}$ to be multivariate standard normally distributed. Thus, the network weights and biases are also normally distributed,

(6) $$\begin{array}{ccc} W_{i} & ∼ & N({\underline{\mu }}_{wi},\;\tau_{wi}^{2}\;\cdot diag\left({\underline{\mu }}_{wi}\right)) \end{array}$$

(7) $$\begin{array}{ccc} B_{i} & ∼ & N({\underline{\mu }}_{bi},\;\tau_{bi}^{2}\;\cdot diag\left({\underline{\mu }}_{bi}\right)). \end{array}$$

The diagonal covariance matrix of the Gaussian variational distributions assumes the independence of all the network parameters and biases. The calculation of the respective gradients and an extension to a tridiagonal covariance matrix can be found in [44] and [52]. We implement the Bayesian segmentation network using the open source Python library PyTorch [53].

### 3.2. Pose Estimation

In order to generate a static simulation model of a complex environment, the pose of each object within the segmented point cloud needs to be determined. Directly fitting objects to the point cloud using for example a “Random Sample Consensus” (RANSAC) [54] approach produces poor results due to the segmentation inaccuracies and intra class variations of the objects. Therefore, pose estimation is carried out for each class j,j∈{1,⋯,m} separately using a clustering approach, which identifies the number of objects within each class and the points belonging to the individual objects. Technically, any clustering algorithm can be applied, however, based on the data set characteristics it makes sense to determine what algorithm suits our use case best. Thus, we compare the classical clustering methods *k*-means [55] and fuzzy *c*-means [56], the density based approaches “Density-Based Spatial Clustering of Applications with Noise” (DBSCAN) [57] and “Ordering Points To Identify the Clustering Structure” (OPTICS) [58] as well as spectral clustering [59] in Section 4.2.4.

After knowing the number of objects within each class their pose needs to be determined. A prerequisite to tackle this problem is the availability of 3D CAD models or at least reference point clouds of the object classes of interest. A point cloud can be sampled from these CAD models resulting in a “perfect” point cloud of an object within class *j*. Iteratively an initial object pose is determined for every object within each class by coarsely registering the “perfect" or reference point cloud and one object of the segmented point cloud using the global registration algorithm RANSAC, which does not require an initial alignment. A tight alignment of the point clouds is achieved using the local registration algorithm ICP that interprets the output of the RANSAC algorithm as an initial alignment. Based on the resulting transformation matrix the respective (x,y,z)-coordinates and the roll, pitch and yaw are calculated for each of the objects. These values are retained for later placement within a simulation environment. Object details should be retained in a way that allows a large number of people to use the data, that is, a holistic data storage and format is needed. The Automation Markup Language (AML) format, which is an open standard to exchange factory engineering data, is a form of meaningfully grouping and ordering the data. Thus, it seamlessly fits into a holistic data model. A pseudo code of the pose estimation step is provided in Algorithm 1. In the pseudo code we assume that the clustering algorithm is capable of determining the number of cluster centres itself. Otherwise, the number of clusters has to be passed to the algorithm as input parameter. Further, we assume the availability of CAD models out of which reference point clouds are generated.
**Algorithm 1:** Pose estimation(m,pc_seg,CAD_models
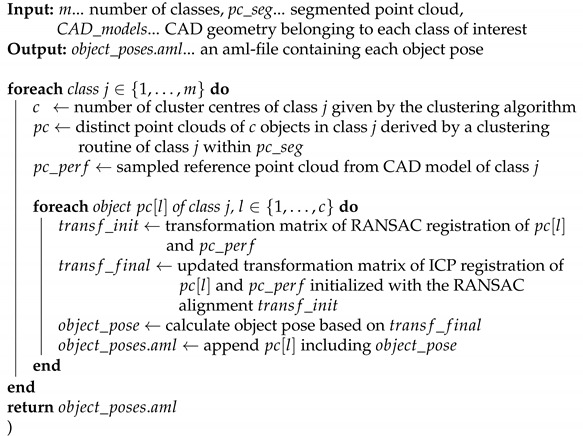


### 3.3. Simulation Model Generation

The extracted object poses can be used to generate a simulation model of the digitalized environment in any simulation tool. Prerequisite for the simulation model generation is the availability of the CAD models of every class of interest and a simulation project having access to these CAD geometries. Of course, also point clouds of objects, which are not available in CAD, can be included in the simulation model. However, as point clouds are not complete, that is, they have holes due to occlusions during the digitalization process, the informative value of the simulation can suffer. For instance, collision checking is not reliable under the presence of potentially incomplete point clouds. Using the positional and rotational data saved in the *object_ poses.aml* file the CAD object is placed in the scene. The placement itself in a simulation tool is straight forward. As a result, a static simulation model is created, which is ready for animation by the user. The logical connection between the objects is supplied by the AML file. It includes the hierarchical order of the objects, the kinematics specifications and the relative positions of related objects in the hierarchy, for example, the relative position of screws within an assembled product. All of this information can be imported in the simulation environment. However, any later changes in the object topology have to be adjusted manually.

In order to display the final environment model generated by our approach, we choose the simulation framework Unreal Engine 4 (UE4) [60] as this simulation tool is widely applied in different industries due to its Blueprints visual scripting system, which is easy to use even for people without software engineering background. Nevertheless, it is also possible to develop simulations using common programming and scripting languages like C++ and Python.

## 4. Results and Analysis

In this section the proposed environment modelling approach is applied to an automotive manufacturing plant. We collect an automotive data set according to the methodologies described in Section 2 and analyse its properties. The automotive factory data set is used for the evaluation of the data processing steps. In the sequel the results and recommended actions for our proposed modelling approach are presented. The Bayesian neural network itself is evaluated in our previous work [10]. Thus, we will briefly summarize the main results for the automotive factory data set and evaluate the remaining process steps in more detail.

### 4.1. Evaluation Data Set

**Figure 4:**
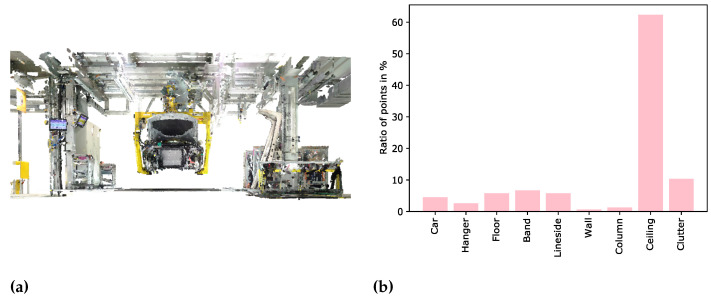
Summary of the generated point cloud. (**a**) One example tact of the collected data set. (**b**) Highly imbalanced class distribution.

The complete environment modelling approach is evaluated on the data set we collect according to the presented methodology, which includes the evaluation of data collection, segmentation and pose estimation. In order to collect the data set, we use a static Faro Focus3D X 130HDR [61] laser scanner, a Nikon D5500 [62] camera with an 8 mm fish-eye lens as well as a Sony Alpha 7R II [63] with a 25 mm wide-angle lens to capture the environment. On the basis of several static laser scans as well as thousands of camera images we generate local laser scan point clouds as well as photogrammetric point clouds for 13 tacts of car body assembly. These local point clouds are registered using circular registration targets generated by the software tool RealityCapture [64]. Initially, the registered global point cloud comprises over one billion points before being cleaned using noise reducing filters. The remaining noise points are removed during the labelling steps using the open source software tool CloudCompare [65]. The final point cloud comprises 594,147,442 points, which corresponds to a removal of about 40% of the points compared to the raw point cloud. The majority of the noise points are the result of motion blur that occurs during scanning and reflections of shinily painted objects. In total the point cloud is divided into 9 classes comprising 8 object categories relevant for factory planning as well as the additional class of clutter, which serves as a placeholder for all the other objects in the scene. The 8 object classes correspond to car, hanger, floor, band, lineside, wall, column and ceiling. These are relevant for factory planning as they are difficult to move or even immovable. One example tact of the collected data set is illustrated in Figure 4a. After labelling all the data, the class distribution can be analysed. We see that the classes are highly imbalanced, which is depicted in Figure 4b.

It is clear that a large ratio of points is assigned to the class ceiling, whereas the classes of wall and column comprise relatively few points. The size of the class ceiling stems from the layered architecture of this structure. In response to this, points on various levels belong to this class. In contrast to that walls and columns are hung with clutter objects like information signs, fire extinguishers or rubbish bins. Therefore, only few points truly belong to these classes and their point clouds have many holes due to occlusions caused by the clutter objects.

**Figure 5:**
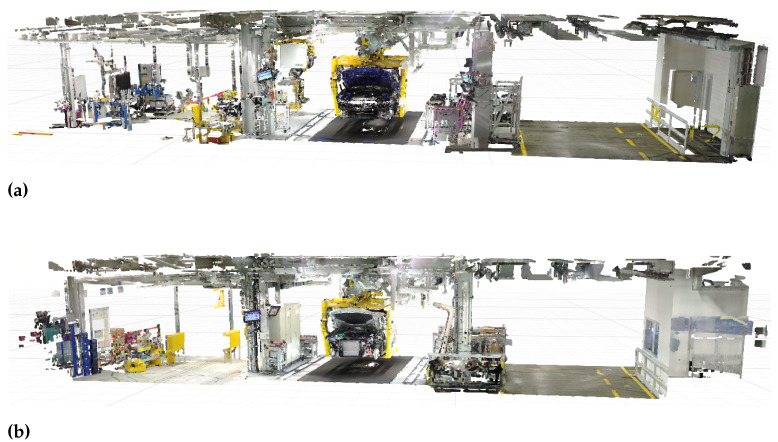
The two test tacts of the automotive factory data set. (**a**) First test tact displaying a blue vehicle to be manufactured. (**b**) Second test tact illustrating a white vehicle later in the assembly process.

Out of the 13 digitalized assembly tacts two are set aside for evaluating different steps of our approach. The remaining tacts are used for model training and validation. Figure 5 displays the two test tacts.

### 4.2. Results

The analysis of the proposed approach starts with evaluating different digitalization solutions in terms of point cloud accuracy, completeness and point density. As mentioned before the evaluation of the Bayesian segmentation network is only summarized. Additionally, different clustering algorithms are analysed for their suitability to our use case. This analysis takes into account the clustering accuracy and time. The accuracy of the pose estimation step is evaluated with respect to the mean deviation of the generated simulation scene to the reference point cloud.

#### 4.2.1. Data Collection and Visualization

For the evaluation of our modelling approach, we first collect the automotive factory data set and analyse the goodness of the resulting point cloud with respect to its accuracy, completeness and point density. In order to calculate the global accuracy of the measured point cloud we suppose that the distance of the measured points to the reference model is normally distributed with mean zero. The variance is given by

(8) $$\sigma^{2}\;=\;\frac{1}{n}\sum_{i=1}^{n}{\left(d_{i}-0\right)}^{2}\;=\;\frac{1}{n}\sum_{i=1}^{n}d_{i}^{2},$$

where $d_{i}$ corresponds to the measured distance of point *i* of the measured point cloud to the reference model. The standard deviation σ is the square root of the variance and is referred to as accuracy of the point cloud, that is, a lower standard deviation corresponds to a higher accuracy. In order to calculate point cloud completeness, the minimum distance of each point in the measured point cloud to the reference model is calculated. Then we define completeness as the percentage of points with a maximum distance of 10 mm to the reference point cloud. The point density is quantified by the mean number of points within a 10 mm radius of each point in the measured point cloud.

We generate point clouds from varying sources, that is, four laser scans per tact, six laser scans per tact, photogrammetry only using a fish-eye and a wide-angle lens, a combination of four laser scans with photogrammetry as well as six laser scans with photogrammetry. The evaluation results for the point clouds generated from these sources are summarized in Table 3. The unit of point density is the mean number of points.

We see that the laser scan only point clouds have a higher accuracy, which corresponds to a lower standard deviation, than point clouds including photogrammetry information. In contrast to that, completeness and point density increase considerably when the photogrammetric information is added to the laser scans. Surprisingly, the point density is highest for the photogrammetry only case using the wide-angle lens. Further, the point density is higher in the case of four laser scans and photogrammetry compared to six laser scans and photogrammetry. Both of the effects are dependent on the number of blurred images that are removed and the quality of image registration. The more images are used for generating the photogrammetric point cloud the higher the resulting point density.

**Figure 6:**
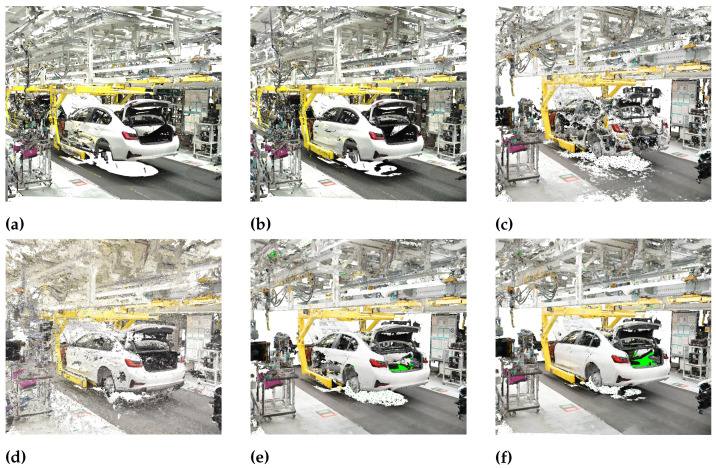
Point clouds resulting from different data sources. (**a**) Four laser scans. (**b**) Six laser scans. (**c**) Photogrammetry with wide-angle lens. (**d**) Photogrammetry with fish-eye lens. (**e**) Four laser scans and photogrammetry. (**f**) Six laser scans and photogrammetry.

For the generation of a simulation model the point density is neglectable as the point clouds are down-sampled before segmentation for efficiency reasons. However, a high accuracy is important in order to ensure correct model placement. Thus, there are different recommended processes for data collection depending on the intended use of the point cloud. Figure 6 shows all the point clouds generated from the given data sources. In Figure 6a,b only the static laser scanner is used, taking four and six scans per tact, respectively. The two purely photogrammetric point clouds are illustrated in Figure 6c,d, which we generate using the wide-angle and the fish-eye lens. The point clouds generated by four and six laser scans complemented with the information of the photogrammetric point cloud are shown in Figure 6e,f. For the combined laser scan and photogrammetric point clouds the wide-angle lens is used.

From a visual inspection, we can see that the point cloud using six laser scans is better in terms of completeness, especially at the side of the car where reflections cause holes in the point cloud generated from four scans. Similarly, the photogrammetric point clouds suffer from reflections due to the shiny car paint. Further, the photogrammetric point cloud generated with the fish-eye lens is distorted, which can also be seen in the higher standard deviation. Generally, images that are taken using a fish-eye lens are easier to register due to a higher number of features being captured on the image. However, cameras with a fish-eye lens are more difficult to calibrate due to the high degree of distortion and the resulting point cloud has a lower accuracy compared to the wide-angle lens. The combined laser scan and photogrammetric point clouds look best in terms of completeness whereby again the point cloud produced from six laser scans has a higher quality.

**Figure 7:**
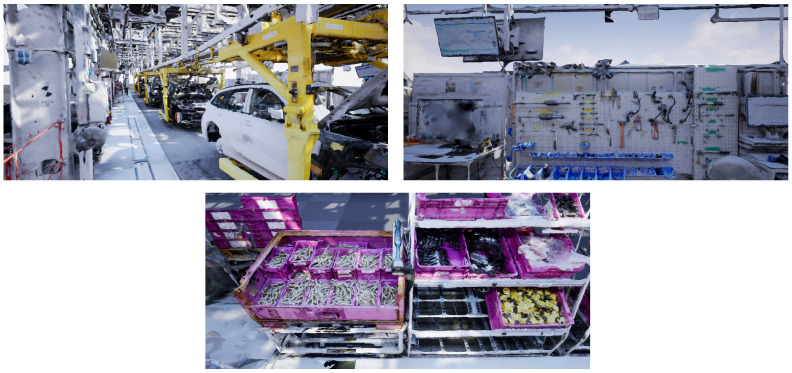
Visualization of the combined laser scan and photogrammetric point cloud as mesh.

The software tool RealityCapture [64] can be used for quickly visualizing the generated point cloud data as a triangle mesh. In order to improve the handling of the mesh, the number of triangles is reduced in Blender [66]. The visualization of the optimized meshes is depicted in Figure 7. The displayed meshes are blocks in which objects are not separated from each other. Thus, they are not directly usable for factory or process simulation since single objects can neither be handled nor animated. However, these visualizations serve as a good starting point for factory planners that are not on-site to get an overview of the current plant layout. Note that we do not aim at creating photo realistic meshes out of point clouds in this work. This process involves high efforts in modelling and texturizing the scene.

#### 4.2.2. Bayesian Segmentation

After generating a global point cloud of the digitalized environment, the segmentation step can be evaluated. To this end, our Bayesian formulation of PointNet is compared to the original PointNet implementation of [15] based on TensorFlow [67] and our PyTorch [53] implementation of frequentist PointNet. The input point clouds comprise assembly tacts, which are cut into equally sized blocks. The number of points within these blocks is sampled to 4096.

The models are trained using mini-batch stochastic gradient descent with a batch size of 16. The momentum parameter is set to 0.9 and a decaying learning rate lr is used with an initial learning rate of lr=0.001 in the frequentist and lr=0.01 in the Bayesian case. The learning rate is decayed every 10 epochs by a factor of 0.7 during frequentist training and 0.9 during Bayesian training. The batch size and the learning rate are optimized by using grid search and cross-validation. In terms of approximation of the posterior predictive distribution, we draw K=50 Monte Carlo samples.

All models converge and the training is stopped after 100 epochs. Table 4 displays the evaluation results of the original PointNet, the frequentist PyTorch implementation and Bayesian PointNet on the test tacts of the automotive factory data set. Bold table entries represent best model performance.

It can be seen that Bayesian PointNet clearly outperforms the original implementation as well as the classical version in terms of segmentation accuracy and the mean intersection over union (IoU) for both data sets. For a more detailed review and interpretation of the segmentation results see [10].

#### 4.2.3. Uncertainty Estimation

The different methods used for uncertainty estimation are described in Section 2.2. They include entropy based estimation methods, the predictive variance as well as the estimation of credible intervals on the network outputs. Each of these quantities are calculated on the basis of K=50 forward passes through the network each using a different weight sample drawn from the variational distribution. Predictive and aleatoric uncertainty are estimated using the Equations (1) and (2). Epistemic uncertainty is the difference of these two values. In order to determine the predictive variance, the unbiased estimator of the variance is used. In the credible interval based method we determine the 95%-credible intervals of the predictive network outputs after normalization with the softmax function.

In order to determine the impact of uncertainty on the network performance, we compare the accuracy of the Bayesian baseline to the accuracy achieved when only considering certain points with respect to all the discussed uncertainty measures, that is, uncertain points are dropped. The results for both test tacts of the automotive factory data set are summarized in Table 5.

It is clear that considering any of the uncertainty estimation approaches considerably increases the segmentation accuracy, with aleatoric and predictive uncertainty performing best. For the entropy based measures as well as the predictive variance a threshold needs to be set, in order to classify a prediction as either certain or uncertain. In this case we consider predictions as certain when the respective uncertainty value is smaller or equal to the mean uncertainty plus two sigma. For the credible interval based method, predictions are considered certain if the 95%-credible interval of the predicted class does not overlap with the 95%-credible interval of any other class. In Table 6 the ratio of points that are considered uncertain by the respective methods are displayed.

Due to the choice of the uncertainty threshold about 4% to 7% of the predictions are dropped using predictive, aleatoric and epistemic uncertainty as well as the predictive variance. In the case of the credible interval based method the ratio of discarded predictions is considerably lower as there is no explicit uncertainty threshold to be set, which leads to a lower segmentation accuracy. However, dropping too many predictions can cause the loss of building or facility structures.

In order to increase the segmentation accuracy, the methods of predictive and aleatoric uncertainty are most promising. Generally, epistemic uncertainty decreases after thorough model training, thus, the predictive uncertainty value is mainly determined by aleatoric uncertainty. In response to this, they lead to similar results. We will use the concept of predictive uncertainty for our upcoming observations and analyses as the credible interval based method does not allow us to control the ratio of dropped points. Further, predictive uncertainty combines the information from aleatoric and epistemic uncertainty, which is more favorable than working solely with aleatoric uncertainty. The remaining uncertainty measures display a lower performance on our data set.

**Figure 8:**
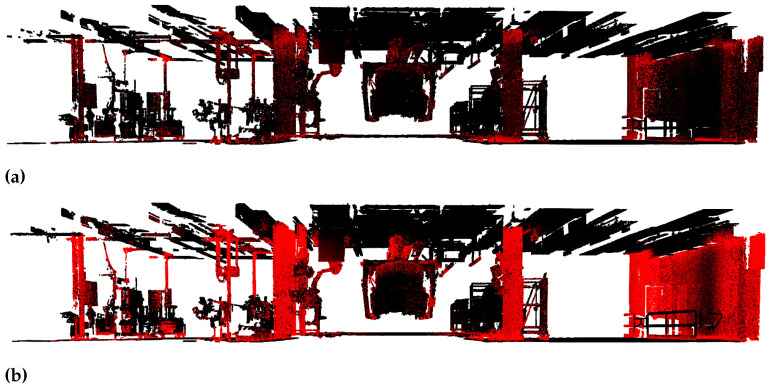
One of the test tacts in the automotive factory data set, where certain points are illustrated in black and uncertain points are illustrated in red. The uncertainty is measured using the predictive uncertainty. (**a**) The uncertainty threshold is set to the mean plus three sigma. (**b**) The uncertainty threshold is set to the mean plus one sigma.

Figure 8 illustrates the importance of being able to control the ratio of dropped points. It shows one test tact of the automotive factory data set. In Figure 8a the uncertainty threshold is set to the mean predictive uncertainty plus three sigma. About 3.24% of the points are classified as uncertain and therefore displayed in red. In Figure 8b the uncertainty threshold is the mean predictive uncertainty plus one sigma. This time about 10.69% of the points are classified as uncertain. It can be seen that important structures like columns and walls are almost entirely classified as uncertain and thus dropped. This is in line with our expectations of Section 4.1 as the classes of wall and column suffer from a high number of holes since they are draped with clutter objects. However, this is disadvantageous when building up a factory model as important structures are missing. Therefore, it is important to find a trade-off between achieving a higher accuracy and dropping a small number of uncertain points.

#### 4.2.4. Pose Estimation

After point cloud segmentation the pose estimation and simulation generation steps are carried out. For pose estimation the number of objects within each class needs to be determined as well as the points belonging to each of these objects. As we already explained in Section 3.2 a clustering routine is applied to tackle these problems. Therefore, we compare the performance of different clustering algorithms including *k*-means, fuzzy *c*-means and spectral clustering. Further, we look into the density based clustering approaches DBSCAN and OPTICS. Table 7 summarizes the evaluation of the clustering algorithms for the classes of car and hanger. These are the two most difficult classes to place as they are highly asymmetric. The building parts are much easier to identify and fit. The table contains the number of points that are classified as the respective class after point cloud segmentation and down-sampling. Further, it illustrates the percentage of points that are assigned to the wrong cluster by each of the algorithms, the percentage of points for which the algorithms are uncertain about the cluster assignment as well as the runtime of the clustering routines. The runtime is determined on a standard office notebook without any specific high-performance components. It can be seen that the clustering methods of *k*-means and fuzzy *c*-means are faster compared to the density based methods and spectral clustering. However, they achieve this performance only when providing them with the exact number of clusters, which makes them impractical for an automated solution. OPTICS and spectral clustering outperform DBSCAN in terms of clustering accuracy but have a higher computational time compared to DBSCAN. Thus, it is dependent on the specific use case, which of the algorithms should be applied. We decide for OPTICS as it determines the number of clusters automatically, is more accurate than DBSCAN and considerably faster than spectral clustering.

As already mentioned, factory layouts are often outdated especially in the case of older plants, thus, we rather compare the object positions of our generated simulation model to the labelled ground truth point cloud than to a plant layout. We place the cars and hangers of the two tacts in the test set in a simulation model based on the ground truth information and retain the gained object poses. We generate the object poses based on the segmented point cloud automatically using the frequentist PointNet (F), Bayesian PointNet (B) and Bayesian PointNet retaining only the predictions that are certain according to predictive uncertainty using the mean uncertainty plus two sigma as a threshold (B+U). We calculate the mean difference between the object poses gained from the ground truth and the mentioned models in terms of the x-, y- and z-coordinate as well as the roll, pitch and yaw between the two sets of object poses. As the placement for the frequentist model is very messy with this approach, we have to add additional information like a minimum height of one metre for each of the objects in order to achieve realistic placement in the scene. The Bayesian network does not need this additional information. Table 8 summarizes the evaluation results of this placement.

It has to be noted that the frequentist model performs best in terms of placement accuracy, however, it requires the input of further object information for proper model placement. In contrast to the frequentist model, the Bayesian model and the Bayesian model with additional uncertainty information achieve similar placement accuracy independent of the additional information, which happens due to the structure of the wrongly classified points in the frequentist and the Bayesian model. The frequentist model tends to misclassify bursts of points. In response to this, the clustering algorithms potentially find more clusters than what are present in real life. The condition of objects having a minimum height of one metre discards these clusters. In contrast to that the Bayesian neural network misclassifies fewer points, which rather tend to be isolated and distributed over the scene. Therefore, the correct number of clusters is found by the clustering algorithms and an additional constraint does not change this outcome. Unexpectedly, the placement accuracy achieved by Bayesian PointNet is even better than in the case of Bayesian PointNet including the uncertainty information. Clearly, the model incorporating uncertainty has a higher segmentation accuracy, however, it discards about 6 % of the points. Thus, it might happen that prominent features that are vital for object placement get lost.

**Figure 9:**
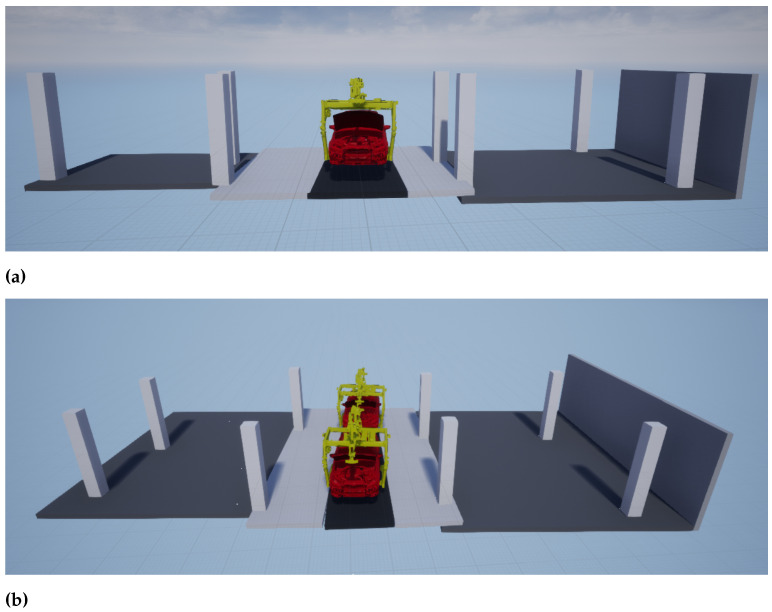
The simulation model generated by our modelling approach displayed in the UE4. (**a**) Simulation model of one test tact. (**b**) Simulation model when both test tacts are processed simultaneously.

The final simulation model for one test tact is depicted by Figure 9a. In this simulation model the ceiling is not displayed for clearness of illustration. Further, the class of clutter is not represented as it is irrelevant for planning tasks. Figure 9b displays both test tacts processed together to generate a single simulation model.

## 5. Discussion and Conclusion

Aside from the presented data collection approaches further digitalization techniques are currently under investigation by the authors to make the process more efficient. In the following, we discuss the challenges and possibilities of digitalization and the generation of factory models. The economic potential of automating the digitalization process and the process of model generation is highlighted for a number of exemplary plants. Finally, a brief conclusion completes our work.

### 5.1. Discussion

In the presented modelling approach plant digitalization is carried out during production free times, which has the advantage of the assembly line standing still and hardly any people being in the plant except maintenance workers. Due to privacy protection regulations, the faces of people that are captured by the laser scanner or the cameras have to be disguised, which is easier during production free time, as fewer people are in the building. However, personal data displayed on process boards and shift schedules still needs to be handled. In this work we collect our data using stationary laser scanners, which usually take some time to capture their surroundings. Thus, in practice it is advisable to use a combination of mobile and static laser scanners, which can still be complemented by photogrammetry if necessary. The plant is ideally digitalized using a mobile laser scanner in order to save time. Only in places where higher accuracy is needed the mobile laser scan can be complemented with a stationary one, for example, when accurate height information plays an important role. In addition, drone technologies are investigated for digitalization purposes, however, the risk of a drone falling down and hurting employees or damaging assembled customer products has to be addressed. Further, we look into technologies using only cameras to generate a 3D model. Generally, these methods are useful for creating a quick and rough overview of the plant. Yet, they are inadequate for most of the planning tasks due to a comparatively low accuracy.

Another consideration is to place laser scanners on a fleet of vehicles that are routinely used within production plants like forklifts, autonomous platforms or hangers on the production line. An incremental model of hundreds or even thousands of laser scans could be built. However, this approach causes scan activities to be carried out during the running production. On the one hand, several challenges like the registration of a high number of laser scans created by different mobile scanners, attached to different vehicle types at different heights and moving with different speeds arise. On the other hand, changes to the facility are detected quickly. Yet, not all the changes are relevant for planning projects. For instance, changes to the building structure are important for planning tasks in terms of spatial restrictions. In contrast to that the different parking positions of a forklift are irrelevant for factory planning. After the detection of a relevant change, a partial update of the factory point cloud is favorable, that is, only the area where a change is detected is updated, in order to keep the computational and storage overhead as low as possible.

At the moment, static simulation scenes are built from laser scans, which correspond to a snap-shot of the production plant at the time of data collection. In future, it is thinkable to generate animated simulation scenes based on a stream of input data in certain parts of the plant, that is, process simulations are generated automatically based on periodical digitalization and data collection. For the time being, this will only be possible for smaller parts of the plant, where special sensory equipment is installed. In order to generate process simulations, the frequency of laser scans must be high. Thus, an efficient scanning strategy is a prerequisite.

The transition from 2D to 3D planning introduces a lot of changes in the IT and process landscape that are necessary for various planning tasks. Hence, dedicated measures of employee training need to be provided in order to support a smooth transition. Generally, the whole process from data collection over final storage to the management of access rights needs to be detailed. Further, an optimized database for point cloud storage, updating and versioning is needed. Software products that are apt for point cloud streaming could be connected to this database as they allow for efficient visualization of the point clouds even on resource constrained devices.

The potential that lies in automating the factory digitalization process and model generation is huge. On the basis of quotes from three different service providers, we conclude that laser scanning of an assembly plant costs about 1–4 € per square metre depending on how much surface geometry is modelled into the scan, thus, we calculate with a low degree of modelling resulting in 1.50 €/m^2^. Assuming an average area of 950,000 m^2^ per production plant we calculate the potential yearly savings of 10 plants. Further, we assume that about 60% of the total plant area is relevant for assembly and needs to be digitalized. We aim at digitalizing a plant once a year and target a degree of automation of about 70%. A higher degree of automation would of course be favorable, however, there are certain aspects that can hardly be automated. For instance, monitoring the digitalization process, checking on the registration targets and the quality of registration will still be handled manually. The above-mentioned assumptions result in a yearly saving of about 6 million Euros. Table 9 summarizes the calculation. Note that the numbers in the above example are hypothetical and do not reflect the real volume and number of the automotive production plants under investigation in this text.

Thus, the economic potential of automating the digitalization process is big. However, these are only the obvious savings gained by the automation of data collection. The availability of a comprehensive digital 3D model of big parts of the assembly area, which reflects the current state of the plant, holds an even bigger potential, when it comes to planning tasks. As already mentioned, planning mistakes can be found in the digital model rather than during the implementation of the changes, which saves a tremendous amount of money and potentially prevents production downtimes. Further, travelling efforts of planners are reduced considerably, thus, they are relieved from this additional burden and can focus on their core business.

### 5.2. Conclusions

In this paper, we describe a holistic and methodical approach to generate a static simulation model in a partially automated fashion from a point cloud of a factory environment. We start with the collection of point clouds in large-scale industrial production plants using laser scanning and photogrammetry techniques. Further, the data pre-processing steps of point cloud registration, cleaning and labelling are described. The data processing steps of segmentation, pose estimation and the final simulation model generation are discussed in detail. For point cloud segmentation the application of a Bayesian neural network is evaluated as it allows the quantification of uncertainty in the network predictions. To this end, three entropy based uncertainty measures as well as the predictive variance and a credible interval based method of uncertainty estimation are discussed.

An automotive factory data set is collected in order to evaluate the digitalization strategy. The different technologies and hardware equipment are evaluated with respect to the resulting point cloud accuracy, completeness and point density. Based on this evaluation we conclude that laser scan point clouds are more accurate than photogrammetric point clouds but they have a lower completeness and point density. Further, the use of wide-angle instead of fish-eye lenses for photogrammetry is highly recommended as the former outperforms the latter with respect to all the evaluation metrics. Thus, in terms of data collection we recommend to combine the point clouds generated by laser scanners and photogrammetry techniques.

The subsequent steps of the proposed modelling approach are evaluated on the collected data set as well. The Bayesian segmentation network clearly outperforms the frequentist model even without considering the additional information provided by the uncertainty in the network predictions. Taking into account the network’s uncertainty in its predictions and evaluating the segmentation accuracy only on certain points increases the network performance considerably using any of the described uncertainty measures. In this case uncertain predictions are discarded. The notions of predictive and aleatoric uncertainty lead to the best results. In the given use case, we decide to focus on the measure of predictive uncertainty as it allows us to control the ratio of points dropped by setting an uncertainty threshold, which is not the case for the credible interval based method. As predictive uncertainty combines the information of both aleatoric and epistemic uncertainty, we prefer this measure over aleatoric uncertainty alone. Based on the segmented point cloud we estimate the poses of relevant objects using, among other methods, clustering techniques. Therefore, the common clustering algorithms *k*-means and fuzzy *c*-means, the density based methods DBSCAN and OPTICS as well as spectral clustering are compared. Even though *k*-means and fuzzy *c*-means clustering achieve superior performance when knowing the number of clusters in advance we advise not to use these methods for an automated solution. All the remaining methods automatically determine the number of clusters. The OPTICS algorithm provides the best trade-off between runtime and clustering accuracy. Thus, we apply this algorithm for the final evaluation of the placement accuracy. The object placement is carried out on the basis of the segmented point cloud using the frequentist and the Bayesian neural network. Additionally, the Bayesian neural network including uncertainty information is used for object placement. The placement in the frequentist case is by far the worst, not producing any reasonable simulation scene without adding additional hand-crafted constraints for object placement. Astonishingly, the placement accuracy is highest for the Bayesian neural network not considering network uncertainty, even though the segmentation accuracy is considerably improved by incorporating uncertainty information. Most probably, the reason is that by considering network uncertainty relevant features of the point cloud are classified as uncertain and thus dropped. Therefore, we conclude that the Bayesian neural network without removing uncertain points is best suited for our use case.

In summary, the major advantage of the proposed modelling approach is the ability to reconstruct a factory environment on object rather than on building level, which extends state-of-the-art reconstruction techniques. Further, this approach does not necessarily require the availability of CAD models of the objects in the scene like the majority of frameworks do. However, the reconstruction results are more reliable under the presence of CAD models. Additionally, the input point cloud is partitioned into meaningful subsets of points early in the process, which allows us to process subsets of points aside from simulation generation within the respective departments.

The proposed modelling approach is applied in a real-world industrial prototype that aims at the reconstruction of automotive assembly plants on the basis of 3D point clouds. On the process side, this paves the way for an automated target-actual comparison of plant assets. For instance, layouts and construction measures can be verified automatically on the basis of the generated factory model. Further, the differences in the environment between two arbitrary digitalization dates can be determined, which is favorable to indicate changes in the building or process structure. These points will be covered in future research. Additionally, the generation of an asset library is supported by our approach, as objects for which CAD models are unavailable can be extracted from the point cloud and their surface can be reconstructed. Currently, this reconstruction is automated for geometrically simple objects like walls or columns. In future work, we study the automatic reconstruction of more complex process related structures. With respect to mathematical concepts, the suitability of uncertainty information for removing noise in point clouds will be analysed. Further, the parameters of the prior distribution in the applied network will no longer be treated as hyper parameters during network optimization. They will rather be estimated in a Bayesian way by the definition of a separate prior and calculation of their posterior.

## Figures and Tables

**Table 1:** Major differences between greenfield and brownfield planning.

Greenfield Planning	Brownfield Planning
Planning of a new plant 3D building plans 3D CAD models of machines and tools Up-to-date data Decide for technologies to be used Regular digitalization taken into account Learn from mistakes in simulations	Renovation of an existing plant Often only 2D building plans 2D construction drawings Often outdated data Live with the technologies given Difficult to digitalize Live with mistakes of direct implementation

**Table 2:** Major differences between stationary and mobile laser scanning.

Stationary Laser Scanning	Mobile Laser Scanning
Fixed on a stationary tripod Higher point cloud accuracy Longer range, which is especially important for height information Inhomogeneous point density and many occlusions due to stationarity Duration of the scan is several minutes	Fixed on a mobile platform Lower point cloud accuracy Shorter range, which is insignificant in the plane More homogeneous point density and fewer occlusions due to scanner path Considerably lower scan duration

**Table 3:** Evaluation of the collected point cloud in terms of accuracy, completeness and point density.

Data Source	Accuracy	Completeness	Point Density
4 Scans	±5.3 mm	41%	89.4
6 Scans	±4.8 mm	51%	146.7
Photogrammetry only wide-angle	±5.8 mm	61%	854
Photogrammetry only fish-eye	±7.8 mm	60%	154.2
4 Scans and Photogrammetry	±6.3 mm	61%	804.3
6 Scans and Photogrammetry	±6.2 mm	64%	789.9

**Table 4:** Segmentation results of the original, classical and Bayesian PointNet on the automotive factory data set. For every model the training converges and is stopped after 100 epochs.

Model	Training Acc.	Test Acc.	Test mIoU
Original PointNet [15]	96.56%	93.37%	80.03
Classical PointNet	97.66%	94.23%	78.08
Bayesian PointNet	98.65%	**95.47%**	**82.63**

**Table 5:** Evaluation of the discussed methods for uncertainty estimation and influence on model accuracy.

	Baseline	Predictive	Aleatoric	Epistemic	Variance	Credible Int.
**Assembly T. 1**	94.21%	96.63%	**96.64%**	95.54%	95.60%	94.99%
**Assembly T. 2**	94.78%	97.41%	**97.47%**	96.22%	96.22%	95.63%

**Table 6:** Percentage of predictions dropped when excluding uncertain predictions.

	Baseline	Predictive	Aleatoric	Epistemic	Variance	Credible
**Assembly T. 1**	-	6.55%	6.56%	4.09%	3.70%	1.47%
**Assembly T. 2**	-	7.08%	7.17%	4.52%	4.09%	1.84%

**Table 7:** Evaluation of different clustering methods on points that belong to the classes of car and hanger after segmentation using the Bayesian neural network.

Object	# Points	Method	Mistakes	Uncertain	Time
Car	30 555	*k*-means	0.14%	-	0.11 s
Car	30 555	*c*-means	0.14%	0.24 %	0.07 s
Car	30 555	DBSCAN	0%	1.51%	3.81 s
Car	30 555	OPTICS	0%	1.51%	45.64 s
Car	30 555	Spectral	0%	-	299.23 s
Hanger	17 454	*k*-means	1.26%	-	0.07 s
Hanger	17 454	*c*-means	1.27%	1.06%	0.04 s
Hanger	17 454	DBSCAN	5.17%	0.91%	1.74 s
Hanger	17 454	OPTICS	0.91%	5.18%	15.78 s
Hanger	17 454	Spectral	0.99%	-	62.21 s

**Table 8:** Evaluation of the model placement for the classes of car and hanger. The frequentist (F), the Bayesian (B) and the Bayesian model including uncertainty (B+U) are used for segmentation. The evaluation metric is the mean deviation of the (x,y,z)-coordinates and the roll, pitch, yaw of the models generated based on the segmented point cloud and the labelled ground truth.

Object	Net	x-coord	y-coord	z-coord	Roll	Pitch	Yaw
Car 1	F	5.48 mm	1.13 mm	0.45 mm	0.19°	0.00°	0.06°
Car 2	F	9.09 mm	16.55 mm	2.74 mm	0.49°	0.24°	0.31°
Hanger 1	F	3.55 mm	0.82 mm	6.92 mm	0.02°	0.15°	0.07°
Hanger 2	F	18.03 mm	1.41 mm	3.92 mm	0.26°	0.25°	0.01°
Car 1	B	0.61 mm	0.34 mm	1.17 mm	0.13°	0.02°	0.03°
Car 2	B	3.49 mm	7.69 mm	1.39 mm	0.17°	0.28°	0.22°
Hanger 1	B	7.43 mm	6.51 mm	3.53 mm	0.17°	0.28°	0.03°
Hanger 2	B	24.58 mm	13.17 mm	7.01 mm	0.40°	0.50°	0.16°
Car 1	B+U	1.83 mm	6.22 mm	0.49 mm	0.21°	0.07°	0.13°
Car 2	B+U	6.8 mm	58.89 mm	36.59 mm	0.06°	0.52°	0.69°
Hanger 1	B+U	2.04 mm	0.97 mm	2.82 mm	0.04°	0.06°	0.04°
Hanger 2	B+U	68.15 mm	15.92 mm	70.94 mm	0.42°	1.36°	0.38°

**Table 9:** Potential of automating the digitalization process using laser scanners only.

Attribute	Value	Unit
Costs per m^2^	1.5	€
Average area of a plant	950,000	m^2^
Percentage of scanned area	60	%
Number of plants	10	#
Number of scans per year	1	#
Total cost per year	8,550,000	€/year
Degree of automation	70	%
Savings per year	5,985,000	€/year

## Data Availability

Not applicable.

## Abbreviations

The following abbreviations are used in this manuscript:

2D	two-dimensional
3D	three-dimensional
AML	Automation Markup Language
BNN	Bayesian Neural Network
CAD	Computer Aided Design
DBSCAN	Density-Based Spatial Clustering of Applications with Noise
DoF	Degrees of Freedom
ICP	Iterative Closest Points
IoU	Intersection over Union
KL	Kullback-Leibler
OEM	Original Equipment Manufacturer
OPTICS	Ordering Points To Identify the Clustering Structure
RANSAC	Random Sample Consensus
UE4	Unreal Engine 4
VDI	Verein Deutscher Ingenieure (Association of German Engineers)
